# The Structural Invisibility of Outsiders: The Role of Migrant Labour in the Meat-Processing Industry

**DOI:** 10.1177/0038038515616354

**Published:** 2016-09-29

**Authors:** John Lever, Paul Milbourne

**Affiliations:** University of Huddersfield, UK; Cardiff University, UK

**Keywords:** civilising process, invisibility, liminality, meat processing, migrant workers, outsiders, Wales

## Abstract

This article examines the role of migrant workers in meat-processing factories in the UK. Drawing on materials from mixed methods research in a number of case study towns across Wales, we explore the structural and spatial processes that position migrant workers as outsiders. While state policy and immigration controls are often presented as a way of protecting migrant workers from work-based exploitation and ensuring jobs for British workers, our research highlights that the situation ‘on the ground’ is more complex. We argue that ‘self-exploitation’ among the migrant workforce is linked to the strategies of employers and the organisation of work, and that hyper-flexible work patterns have reinforced the spatial and social invisibilities of migrant workers in this sector. While this creates problems for migrant workers, we conclude that it is beneficial to supermarkets looking to supply consumers with the regular supply of cheap food to which they have become accustomed.

## Introduction

During the last three decades there has been a significant shift in power within the UK food industry away from small-scale producers and manufacturers towards large retailers and multinational corporations. As power has been concentrated into the hands of the so-called ‘big four’ supermarkets – Tesco, Asda, Sainsbury’s and Morrisons – competitive pressures between the sub-contracted growers, producers and processers below them in the supply chain have intensified. Large retailers have squeezed profit margins and independent producers have become ‘amalgamated via acquisitions, mergers and cooperatives into large and more specialised industrial operations’ (Geddes and Scott, 2010: 196). This increasing gap between those at the top and bottom of the supply chain has been accompanied by two other trends during recent years. First, there has been a rise in the incidence of low paid, irregular and non-unionised work in the agricultural and meat processing sectors (Rogaly, 2008). Second, and related to the first trend, employment in these sectors has become less attractive to the UK workforce, leading to labour shortages (Geddes and Scott, 2010).

These emerging processes of flexible employment have been subject to critical reflection within economics. Drawing on key ideas within segmented labour market theory, attention has been given to how the use of a flexible workforce in a ‘secondary labour market’ allows companies to meet fluctuating demand by offsetting costs and passing on market uncertainty to groups of *outsiders* employed in poorly paid jobs (Doeringer and Piore, 1971; Massey et al., 1998; Piore, 1979). The enlargement of the European Union in 2004 and 2007 has opened up a new pool of cheap labour from outside the UK (Geddes and Scott, 2010), with a disproportionate number of migrant workers from Central and Eastern European countries now gaining employment in the agri-food sector and the meat-processing industry in particular. From the slaughter of livestock to the production of fresh, chilled and frozen meat products, the work involved in the meat-processing sector – boning, freezing, preserving and packing meat – is widely recognised to be dirty, dangerous, demanding and unattractive to UK workers.

The UK state has positioned migrant workers in very particular ways in recent years (Anderson and Ruhs, 2010; Tannock, 2013). Immigration controls have often been presented as a way of protecting migrant workers from exploitation *and* ensuring jobs remain available for British workers. After European Union enlargement in 2004, for example, new European citizens were only permitted to work in the UK if they joined the Worker Registration Scheme (Anderson, 2010). Employment agencies were significant in this context and from 2004 onwards they played an important role in the recruitment and provision of workers from Central and Eastern Europe to meat-processing factories across Wales. This was beneficial to employers, as:

Agency workers have significantly fewer rights than those who are directly employed; they can be hired on lower hourly rates and on worse times and conditions, and they do not have rights to benefits such as overtime and sickness pay. They are also less likely to be members of a trade union. (Anderson and Ruhs, 2010: 21–22)

What also follows from this is that employers have more control over *desirable* migrant workers than they do over *reluctant* British workers (Anderson, 2010).

This situation creates particular difficulties for migrant workers. A recent inquiry into conditions of employment and working practices in meat and poultry processing factories across England and Wales (EHRC, 2010), found that migrants employed in the sector – particularly agency workers – experienced multiple problems, including management coercion and physical and verbal abuse within the workplace. It also revealed that long hours and shift work spent almost entirely in the company of co-nationals was undermining opportunities to improve English language skills and integrate with local communities (see Ciupijus, 2011). The role of supermarkets within these processes was also emphasised. ‘Just in time’ strategies are now widely used by the ‘big four’ to connect different parts of the supply chain and conditions for food processors are shaped by the competitive pressures generated. Indeed, research demonstrates just how successful supermarkets are at transferring these pressures – most notably ‘risks’ and ‘costs’ – down the supply chain (Newsome et al., 2007). The production of perishable meat products is one of the most labour intensive and least profitable parts of the sector and it is all but impossible to control delivery by keeping levels of stock high (Caroli et al., 2007). As meat-processing factories and other supply chain actors struggle to meet the changing demands of supermarkets for ‘just in time’ production (Miele and Lever, 2013), they look to adopt more flexible labour supply practices, with migrant workers playing an increasingly important part in these new working arrangements (EHRC, 2010; Lloyd and James, 2008).

### The Structural Invisibility of Outsiders

Work inspired by Elias (2012) provides a useful framework to understand the position of migrant workers. Elias argues that the civilising process moves forward through a long sequence of spurts and counter-spurts, as some outsider groups slowly attain the characteristics and functions of an established order. The key point for our analysis of migrant workers in meat-processing factories is that the increase in mutual identification that accompanies this process sometimes bypasses certain groups. De Swaan (2001) suggests that this involves a process of compartmentalisation whereby, in a particular *space* – detached from *place* by a wall of invisibility – cruelty reigns. In extreme cases, under *decivilising* conditions a political regime may ‘mobilize the entire machinery of the state to persecute and annihilate this target group’ (De Swaan, 2001: 268).^1^ However, compartmentalisation works by degrees and it may occur under seemingly innocuous conditions in consumer societies through a process of *dyscivilisation*, where contrasts such as good/bad and moral/immoral may exist in tightly defined spaces (De Swaan, 2001).

Matsinhe (2011) argues that the enclaves of cruelty produced through compartmentalisation can be profitably linked to Turner’s (1979a, 1979b) notion of *liminality*. Turner identifies three distinct phases – or ‘rites of passage’ – through which all outsiders pass: separation from a ‘fixed state’; margin (*limen*); and aggregation to a new ‘position’ or ‘state’. Writing about Mexican nationals in the United States, Chavez (1991) draws on these insights to highlight the conditions foreign nationals experience in their passage from the old to the new. His research indicates that the final stage of incorporation in a new country is rarely achieved, with migrants often remaining marginal members of society. What is interesting for our analysis is the idea that the *liminal* phase is also bound up with spatial as well as structural forms of invisibility.

The structural and spatial invisibility of migrant workers is well documented in Anderson’s (2000) account of domestic labour and care work. Highlighting the physical, mental and sexual abuse experienced by female migrants, Anderson draws attention to a range of issues that are relevant to our analysis of migrant workers in meat-processing factories. Most notable is the stratification of domestic workers into racial, ethnic and national groups, the prevalence of flexible working practices and the problematic nature of trade union membership (Anderson, 2006). Her research also documents the consequences of these practices in relation to ill health and financial debt when migrants lose their job unexpectedly and have no recourse to public funds. The fact that care work largely takes place in the home provides a geographical dimension to its invisibility, with workers operating largely in isolation within private/domestic and dispersed spaces.

## Migrant Labour in the Meat-Processing Sector in Wales

In this article we explore these ideas of marginalisation, exploitation and liminality among migrant workers by drawing on research materials from a recent study of migrants within the meat-processing sector in Wales (UK). Meat processing is particularly important for the Welsh economy, recording an annual turnover in excess of £600 million and accounting for the employment of more than 6000 workers (EHRC, 2010). Our research employed a mixed method approach to examine the situations and experiences of migrant workers across a number of interlinked case studies in Wales. Our primary focus is on workers who have migrated from new European Union member states – the so-called A8^2^ and A2^3^ countries – but our research also involved encounters with migrant workers from southern Europe and the Philippines. We selected case study places on the basis of analyses of available data sets, most notably Worker Registration Scheme (WRS) statistics, as well as a number of contextual interviews with key stakeholders at the local, regional and national levels, and an examination of previous studies of migrant workers in Wales (see Threadgold et al., 2008; Woods and Watkin, 2008). On the basis of this preliminary work, we selected five case study areas that had witnessed the arrival of significant numbers of migrant workers in recent years: Merthyr Tydfil, an ex-industrial town in the South Wales Valleys; Llanelli, a large town located in the south of rural Wales; the small rural towns of Llanybydder and Lampeter in west Wales; Welshpool, a medium-sized market town in mid-Wales; and a string of small rural and coastal towns in North Wales.

There were four phases to our research. First, we analysed data from the Worker Registration Scheme, National Insurance registrations and the 2011 Census of Population to provide more detailed temporal, spatial and social accounts of migrant workers in the study areas. Second, we conducted around 50 semi-structured interviews, with various stakeholders, including representatives of national and local government, voluntary sector organisations, health boards, trade unions and migrant support groups, to provide contextual information on the impacts of economic migration in different parts of Wales and our study areas in particular. Third, with the assistance of local stakeholders, a face-to-face questionnaire survey of 109 migrant workers was undertaken in the study areas. The questionnaire consisted of a series of closed and open questions that sought to collect a broad range of material on migrant workers, including the migratory journey, employment, home and community relations. Our final phase of research consisted of follow-on semi-structured interviews with a small number of migrant workers who had participated in the previous phase of fieldwork in order to explore emerging themes in more depth. Descriptive statistical analysis of the quantitative data from the survey was undertaken using Excel, with the sections of the interview schedule used to structure the analysis. Open-ended responses and the material from the semi-structured interviews were analysed using conventional qualitative techniques of coding and sorting.

Table 1 provides an overview of some key characteristics of the 109 migrant workers involved in our research. In terms of nationality,^4^ Polish migrants were the largest group, accounting for 81 per cent of all migrant workers interviewed. However, there was some variation evident across our study areas. Poles accounted for all migrant workers surveyed in Llanelli, Llanybydder and Lampeter, and Welshpool. By contrast, Merthyr Tydfil also contained migrants from Portugal and the Philippines, and the research in the North Wales study area revealed migrants from several other Central and Eastern European countries. Turning to gender, women formed the majority of migrant workers in the survey, although there was again variation across the study areas, which would appear to reflect local employment opportunities. The age profile of migrants is skewed towards younger groups, with those aged 25–34 years representing the largest group, and around half of all migrant workers aged under 35 years. Lastly, in terms of educational qualifications, the majority of migrant workers possessed post-school qualifications, with 31 per cent holding degrees and 28 per cent having a vocational qualification.

**Table 1.** Selected characteristics of migrant workers in the case study areas.

Area	Nationality		Gender		Age					Highest educational qualification		
	Polish	Other	Male	Female	18–24	25–34	35–44	45–54	55+	Degree	Vocational	School
Llanelli	20	0	11	9	5	6	2	5	2	4	6	10
Llanybydder and Lampeter	28	0	15	13	4	9	5	8	2	3	5	20
Welshpool	14	0	0	14	1	9	3	0	1	6	6	2
North Wales	11	8	5	14	0	7	4	4	4	10	8	1
Merthyr	15	13	12	16	1	14	5	5	3	11	5	12
Total	88	21	43	66	11	45	19	22	12	34	30	45

Our study aimed to examine the situations and experiences of migrant workers from Central and Eastern European countries across different employment sectors. However, our initial analysis of Worker Registration Scheme, survey and stakeholder interview data revealed that workers were disproportionately employed in food-processing, which led us to consider their position in this sector in greater depth. Of those migrants surveyed in Merthyr, more than 80 per cent of Portuguese and Polish migrants worked in the food sector, with 65 per cent in meat-processing factories. In Llanelli, 87 per cent were employed in a local meat-processing factory. In Llanybydder and Lampeter almost two-thirds of migrants worked in one meat-processing factory, while in Welshpool just under half of migrants worked in a local frozen food factory.

### The Road to Nowhere

The compartmentalisation of work in meat-processing factories first became evident during the early days of fieldwork. We were able to access migrants in most places through engagement with local gatekeepers in the community, but the research team encountered many problems attempting to access the management of local factories, and during 12 months of fieldwork we only managed to recruit one individual. The pressures faced by factory management attempting to meet supermarket demands are intense (EHRC, 2010; Lloyd and James, 2008; Newsome et al., 2007) and there was clearly suspicion of researchers seeking access to local factories. Speaking with recruitment agencies was just as problematic and we only managed to speak with one ex-employee during the period of research.

**Figures 1 and 2.**
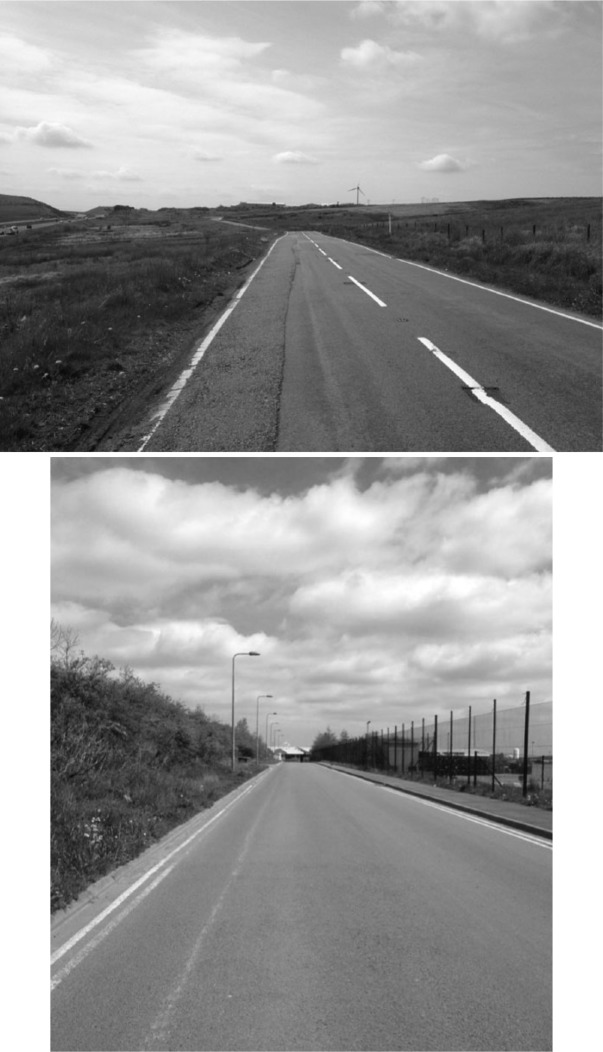
The Road to Nowhere: The approach to a meat-processing factory near Merthyr Tydfil in the Welsh Valleys.

The gatekeepers and research contacts approached to facilitate access were highly aware of the issues involved, with a typical comment being ‘well, I can give you their number, but I don’t think you’ll have much luck’. In the preliminary stages of our fieldwork, there was a strong sense that migrants employed in these factories were outsiders cut off from the rest of society, existing within regimes of labour that were difficult if not impossible to access. The factories they worked within are detached from place in very particular ways, located ‘out of sight and out of mind’ in bland industrial parks on the edge of urban centres, such as Llanelli and Merthyr, or on the periphery of rural towns such as Llanybydder and Lampeter (see also Vialles, 1994) (see Figures 1 and 2).

### Workplace Stratification

Many of the complexities of factory life came into sharp focus when examining the situations of Polish and Portuguese workers in Merthyr. Until 2004, Portuguese workers were the established migrant worker community in the local meat-processing sector. However, almost immediately after European Union enlargement some factories began to use agency-sourced workers from Poland rather than Portugal (Tannock, 2013). In 2005 about 100 out of 900 workers at the major factory in Merthyr were from Portugal, but by 2008 the workforce of 1016 included 448 workers from Poland and 121 from Portugal (MTCBC, 2008; Tannock, 2013). Around half of the migrants from Portugal interviewed in Merthyr arrived for a prearranged job, or because friends or family had suggested that work was available. However, as the number of Polish workers began to increase, tensions between workers from Portugal intensified. As one established male migrant from Portugal commented: ‘Portuguese people, instead of helping and making it easier for the new ones, they feel like they are trying to steal their jobs so they just make everything more difficult.’

Competitive tensions between the Portuguese and Polish groups are strong and workers are often segregated by nationality on production lines to maintain competitive production cycles. One local authority stakeholder confirmed this situation and its consequences in the following way: ‘[s]o you’ll have three lines of Polish workers and a Polish supervisor, two lines of Portuguese workers with a Portuguese supervisor, so there’s no need to communicate with each other’. While such practice clearly lessens the likelihood of migrants learning English and integrating with local communities (Ciupijus, 2011), it was argued that it is difficult to stop if employers find it beneficial.

Arguably these segregated working practices are based on a policy of divide and rule, whereby rivalries between workers of different nationalities are initiated to undermine any gains each group could potentially obtain collectively. This compartmentalised existence in the occupational spaces of the factory floor creates many problems for migrant workers. As they struggle to maintain their position in the hierarchy of factory labour, Polish and Portuguese workers push themselves to physical and mental extremes. Polish workers in particular are valued for their strong work ethic, yet this self-perception often operates as a form of entrapment, normalising a culture of long working hours and intensive working conditions (see Cook et al., 2011; Nowicka, 2013). As a male Polish worker in Merthyr confirmed: ‘this is the reason that the owners are looking for people from Poland […] because they know that they are hardworking people and they are able to work for this money’.

This policy of divide and rule is a strategy employed by numerous employers in a sector where unions have become increasingly peripheral. As in Anderson’s (2000) study of domestic labour, racial, ethnic and national hierarchies feed into wider relations between workers of different nationalities. Over time, recruitment and employment agencies have played a central role perpetuating these differences and wider discourses related to ‘good’ and ‘bad’ workers (Tannock, 2013). Migrants employed in meat-processing factories work long and demanding shifts and they are often on call day and night by agencies and factories alike. A Polish woman employed as a language support worker described the economic uncertainty and insecurity engendered by this situation in the following way: ‘They [migrants] have to be on standby with their mobile phones. They don’t dare to switch it off, even on their day off because they don’t know when the agency may call […] That’s not much of a life.’

It is important to note at this juncture that British workers are often treated in much the same ways as migrant workers, a situation highlighted by a representative of the union UNITE in Powys. Asked how companies running local factories treat migrant workers, our interviewee suggested that ‘they will treat everybody badly if they can, they just don’t care if they are Polish or British or Welsh’.

Since the influx of migrant labour from the European Union began in 2004, the bargaining power of all workers in the sector has been effectively curtailed as widespread opposition to union membership has emerged. Factories across our case studies have successfully kept trade unions out of the workplace and this situation has made it difficult for the unions to deal with claims that migrants are being abused. Although it proved impossible to gain access to workers in the meat-processing sector in North Wales, a trade union representative approached to facilitate access to factories in Anglesey suggested that many companies run a ‘tight ship’ and stay just the right side of minimum legal requirements on any number of issues. He argued that factory owners have fought hard to keep trade unions out of the workplace and that this has had a direct impact on the makeup of the migrant worker population in meat-processing factories across the region.

The impact and use of nationality as a means of stratification was clearly evident in relation to trade union membership. On occasions, Portuguese workers had been dismissed by nationality *en masse* because they were members of a trade union while more recently arrived Polish workers were not. As one local authority stakeholder pointed out: There were […] quite a lot of Portuguese workers, and they got themselves unionised, at which point they were all got rid of and Polish workers were brought in’.

### Employment Agencies, Zero Hour Contracts and Flexibility

Our research highlighted concerns over overtime, wages, holiday entitlement and tied accommodation related to agency employment as being the major problems faced by migrant workers in recent years (see also Jayaweera and Anderson, 2008). As one Polish female worker in Welshpool noted: ‘before I had a contract of employment I worked through agency, and I had problems with getting paid if I was on holiday or when I worked overtime’. While these issues can still be challenging for some workers in particular places, employers have now developed more sophisticated contractual arrangements to manage their workforce. There has been a general move away from temporary agency-based work with many factories now employing migrants on ‘permanent’ contracts. However, this shift is not as straightforward as it seems. In many places the position of migrants as outsiders is maintained by the use of contracts that are often referred to as ‘unlimited’ or ‘opened ended’, but which in reality actually represent ‘zero hours’ contracts that lack any agreed allocation of working hours (see also Watt, 2014).

There were numerous examples of the operation of such working practices across our case studies. In periods when factories were working at full capacity, for example, at Christmas, migrants in Merthyr were required to work 12-hour shifts for weeks at a time or risk losing their job. When orders were scarce, however, they were sent home with no pay and no knowledge of when they would work again. As a couple of migrant workers in Merthyr commented:

If the factories have got a lot of orders they ask us to work seven days a week without any days off and we just have to go to work […] But when the factory didn’t have enough orders we need to stay at home even seven days a week. (Female Polish worker)

I would prefer to work 40 hours […] I don’t work all the days I was supposed to [and] they send me home sometimes. If I worked the four days per week I was supposed to, I would work about 48 hours. (Male Portuguese worker)

Around 65 per cent of migrants surveyed in Llanybydder and Lampeter worked in the meat-processing sector. All but one were based in the same factory in Llanybydder, which in early 2013 had a Polish workforce of 308 out of 577 employees. All worked directly for the employer and described themselves as having permanent contracts. As in Merthyr, the number of hours each migrant worked on a week-by-week basis varied greatly, in this case from 20 to 60 hours.

The use of agencies in the Welshpool area had also decreased in recent years as companies had been able to source staff via word-of-mouth, yet here too it appears that migrants were employed on open-ended contracts. The move towards direct employment was seen by one local authority stakeholder in Welshpool to be a direct response to the specific qualities of Polish workers: ‘I have personal knowledge of their workforce; they would positively employ Polish people. The agency workers are now their permanent workers. They are the best workers and there is no doubt about that.’ A human resource manager at a factory also described the gradual shift away from agency work as a positive trend, both for migrant workers and for companies. However, the conditions of those employed on permanent contracts are ambiguous to say the least, with the widespread use of hyper-flexible arrangements allowing companies to legally end migrant employment at the time of their choosing. Our survey and interviews revealed that while migrant workers often complained about the lack of work and the use of flexible contracts, most accepted their situation with a sense of resignation (Elias and Scotson, 2008; May, 2004). Indeed, most migrants working in factories across our cases studies indicated that the hours they worked and the wages they received were standard for the meat-processing sector. Moreover, across the five areas, 71 per cent of migrants in the survey considered that they were financially better off working in Wales than in their home country.

### Class, Gender and Well-Being

Working-class men once resisted the devaluation of class through the promotion of their physical abilities (Sennett and Cobb, 1972; Simpson et al., 2014). Today the elevation of cultural and gender difference in meat-processing factories across Wales makes this process more complex and difficult to negotiate. There is often an assumption that migrant workers are a homogeneous group, but this is an oversimplification and class, cultural and gender-based differences can be the source of various tensions in the workplace. Despite diverse class origins and cultural differences within the migrant labour force, ‘undifferentiated proletarianisation’ is evident in widespread processes of deskilling and in general patterns of downward social mobility (Virdee, 2014; see also Nowicka, 2012).

Although the majority of our interviewees possessed degrees or vocational qualifications, most had been unable to secure employment that matched their qualification and/or skills (see also Bloch and McKay, 2014). As a Polish male migrant with a postgraduate qualification working as a manual operative in Merthyr noted, ‘to be honest with you, I wasn’t looking for this kind of job’. A small number of migrants did find this situation problematic, but for many others having a job was more important than having a job for which they are qualified. As another over-qualified female Polish worker commented, ‘for me [what is] important is just to have a job, [it] doesn’t matter what kind of field’. That said, we did recruit a small number of skilled migrant butchers in certain places in rural Wales where poor pay had prohibited the employment of British workers. Our research also revealed that a couple of migrants had been promoted to head of a production line comprising migrants of the same nationality, although none of those interviewed were aware of any migrant workers moving into factory management.

In Merthyr and Llanelli around four-fifths of migrant workers were employed as manual operatives in meat-processing factories, a situation that often led to tensions with lesser skilled migrant workers on the factory floor. Several spoke of the need to ‘keep myself-to-myself’ to avoid simple misunderstandings and disagreements. A male Polish worker with a vocational qualification explained how it was necessary to take lunch breaks on his own in order to avoid disagreements with fellow workers from the local area *and* from his home country. As he stated when asked about this situation: ‘I just eat on my own, it’s the easiest way to avoid confrontations.’ Very often these problems were linked to language usage, particularly in Llanelli, Llanybydder and Lampeter, and North Wales where Welsh represented the dominant language of the factory floor. In these places, the language of the workplace acted to reinforce the position of migrant workers as outsiders.

The number of women moving to Wales from Central and Eastern European countries has increased dramatically over recent years. In the immediate post-2004 period, three out of five migrant workers in rural Wales were male (Woods, 2011), but the WRS data indicate that the dominant form of migration across Wales now involves women, both as single people and as partners of the pioneer male migrants. Those moving to be with male partners experienced particular difficulties in areas where the only available jobs were in meat-processing factories. While a small number of females had secured work as teaching assistants or as advisers for migrant support organisations, most had no option but to work on the factory floor in the food-processing sector. Many of the women we surveyed were working below their education or skill level in these factories; this included women with postgraduate degrees in teaching and physics, for example, and a recently arrived bank worker with a degree in economics. Migrant women with children would also support each other with childcare responsibilities to cover the vagaries of hyper-flexible work patterns.

Within the factories, workplace relations appeared to be particularly difficult for female migrants to negotiate, with our research highlighting incidences of verbal and physical abuse. One Polish woman working in a factory in Carmarthenshire commented that she had encountered different forms of abuse from a factory supervisor:

One of the supervisors was verbally abusing Polish workers and I was actually kicked in my bottom by this man […] He was dismissed but the culture of use and abuse of Polish workers as […] cheap labour still exists in that place.

Indeed, several women across our case studies stated they had experienced depression and stress-related illness as a result of their working environment. These issues were particularly acute for those who could not secure familial support in the locality, and/or had no recourse to public funds.

Semi-structured interviews with stakeholders provided further information on the well-being of migrant workers. Several suggested that work in meat-processing factories was linked directly to physical and mental health problems outside the workplace. For example, a representative of the Welsh Polish Mutual Association in Llanelli considered that new arrivals were particularly vulnerable. Some agencies working in the area provided ‘complete packages’ to prospective Polish migrant workers, including travel arrangements and accommodation, training and allocation to a local factory on arrival (see Thompson et al., 2010). However, as many migrants were expecting to begin working as soon as they arrived, any ‘training’ period without pay created financial hardship and a great deal of stress, and we uncovered incidents of migrants turning to petty crime to survive. More generally, interviewees at a welfare rights agency in Merthyr indicated that debt is now a significant problem for migrant workers (see also Anderson, 2000), particularly when they have families to support, much as it is for local workers on low wages and flexible contracts. If migrants are placed on limited hours at short notice, for example, or lose their job, they are often unable to claim benefits because they have not been unemployed or without income for the required period. In this situation, and without recourse to public funds, household finances become stretched and some migrants are forced to rely on doorstep lenders, which can create additional monetary problems.

## Contradictory Discourses of Migrant Work and Migrant Workers

We noted above that UK immigration policy is often presented by national government in terms of the protection they offer migrant workers from exploitation and British workers seeking employment. However, the impact of this policy ‘on the ground’ is far more complex. For example, when a large meat-processing factory opened in Merthyr in the late 1990s, research by Tannock (2013: 11) revealed that ‘suspicions were raised locally that, despite rhetoric of wanting to provide “good” and “well paid” jobs for locals’, the company was coming to Merthyr ‘because Wales was widely seen as a low-wage destination where employers could make more profits’. Tannock argues that government policy and support for the project thus allowed the company running the factory to ‘bully’ local workers until the available supply of local labour dried up. The factory’s owners then turned to Portuguese workers, and later to Polish workers, who were treated in much the same way. A similar pattern of recruitment and working relations is evident in Llanybydder and Lampeter, where Polish, Welsh and English workers are employed.

Contradictory discourses of migrant workers were evident in relation to their positions within and beyond the workplace. Inside the factories migrants were very much valued for their strong work ethic. In some cases this had created competition and tensions between different groups of factory workers, such as the Portuguese and Polish in Merthyr. More generally, the value placed on this work ethic had normalised a culture of long working hours and intensive working conditions for migrants (see also Cook et al., 2011). Beyond the factory, interviews with local stakeholders and migrant workers revealed that migrants remained detached from local society and community. The research revealed incidents of some people in the local area accusing migrants of stealing their jobs. Migrants often linked such a viewpoint to the people they were (seen to be) competing with for local jobs, yet other studies have shown that British workers are often reluctant to take up this type of work and that employers often prefer migrant labour (Geddes and Scott, 2010). As an established migrant worker from Portugal commented:

It’s a crap job, everyone know[s] it […] We haven’t come to steal jobs from anyone, we just come to do jobs that no one else wants to do, and the thing is for us it’s not the job that we want to do as well.

Such narratives not only help to cement the position of migrants at the bottom of the occupational hierarchy, but add to the liminality of their overall experience in communities across Wales.

On leaving a previous fixed state or existence in their home countries, many migrants live on the social fringe as marginal members of society (Chavez, 1991; Turner, 1979b). Hyper-flexible contracts and arduous work patterns mean that they have little time for anything other than work. As a Catholic Priest stated in relation to church attendance: ‘they [migrants] work over the weekends, they work anti-social hours, they work at night time, which means they can’t come to anything […] and they won’t mix’. This situation was confirmed by a recently arrived female Polish worker, who commented that ‘in the factory we work a lot of hours […] so you don’t have a lot of time to do much more’. In addition, some migrants spent several hours each day travelling to and from the workplace. Some factories are not served by public transport and getting to the workplace can be extremely difficult. These difficulties are compounded by the fact that when they arrive at the workplace, migrants – as well as local workers – are often informed that there is ‘no work today’.

Underlying problems of economic marginalisation among the local population have created negative attitudes towards migrant workers rather than focusing attention on the low-wage and low-skill nature of the local economy, and employment-related problems experienced by local *and* migrant workers. Liminality, in this sense, as Turner (1979a: 465) observes, expresses a ‘state or process which is betwixt-and-between the normal, day-to-day […] states and processes of […] registering structural status’. This situation was evident in migrant responses to questions about their relationships with the local community. As a recently arrived Pole in Merthyr noted, ‘it’s not that I’m unwelcome, but also I’m not welcome, they don’t make an effort to make me feel welcome nor the opposite’. Another commented in similar terms: ‘so that feeling of being welcome, it’s like […] I don’t feel welcome but it doesn’t mean that I am unwelcome’.

Migrants rarely provided examples of discrimination and those that were discussed tended to be related to the workplace rather than the local community. A small number of migrants in Merthyr highlighted the dangers of going out late at night in places where young people congregate to drink and socialise. However, these views were in the minority and many migrants involved in the research had positive opinions of local populations, though it should be recognised that few constructed neighbours as close friends or participated in organised community activities. Where negative local attitudes towards migrants were apparent, the perception that migrant workers were taking local jobs was often accompanied, in small rural towns, by observations about the profile and behaviour of previous migrants. This often involved young, single and more transient groups of men, with one housing stakeholder in Welshpool pointing to ‘inappropriate’ drinking cultures in public spaces. In addition, it was apparent that some more settled migrants were now beginning to distance themselves from newer arrivals and seek closer ties with local communities. While these differing associations can be viewed as bound up with the process of assimilation into the host community, it was argued that they also reflect the competition for local jobs and resources between migrant workers.

## Conclusion

In *Utopia* Thomas More (1989 [1516]) discusses the sensibilities that he believed would characterise ‘civilised’ life in the West at some future point. In an insightful passage, More refers to the ‘special places’ outside town where livestock are slaughtered, their carcasses cleansed by slaves to protect ordinary people from moral contamination. Arguably migrants are still positioned in this way – as outsiders – because they perform a similar role for society. Some tasks are essential for society to function successfully, and for these tasks to be carried out effectively, some groups – in this case migrants – *must* be marginalised and positioned as ‘outsiders’ (Copeland, 2007). As we have seen, many migrants accept their situation with a sense of resignation and an understanding that no other group will undertake these tasks. As outsiders with fewer rights than the rest of the population, it is also the case that they are not in a position to question their situation.

In this article we have argued that the process of compartmentalisation is necessary to draw a veil over employment conditions and working practices that help to maintain the cheap supply of food consumers demand and supermarkets supply. As Turner (1979b: 235) comments, ‘as members of society, most of us see only what we expect to see, and what we expect to see is what we are conditioned to see when we have learned the definitions and classifications of our cultures’. Outsiders must therefore remain ‘hidden, since it is a paradox, a scandal, to see what ought not to be there’ (Turner, 1979b: 237). This invisibility is maintained through employment practices and working conditions that divide diverse groups of migrant workers and undermine any degree of solidarity and collective action on the part of the workforce.

As in Anderson’s (2000) account of domestic labour and care work, migrants working in meat-processing factories experience various forms of abuse related to their structural and spatial invisibility. They are physically located in peripheral spaces away from the gaze of the public and the media. Stratified into ethnic and national groups, many are employed on increasingly flexible contracts that require them to be on call day and night. Class and gender create problems for migrant workers and a widespread process of *proletarianisation* and deskilling underpins general downward social mobility (Virdee, 2014). The consequences in terms of pay and exploitation are similar and many migrants end up in debt or ill health, confined to a precarious existence (Anderson, 2010) as ‘outsiders’ on the social fringe. In this article we have argued that this situation is perpetuated, and in some senses enhanced by the use of contradictory migrant worker discourses both inside and outside the workplace. As Chavez (1991: 274) notes, even if migrants ‘imagine themselves as community members, their full incorporation depends not on their own beliefs or actions but, ultimately, on the larger society’s perception’.

Taken together, Turner’s (1979a, 1979b) notion of *liminality* and De Swaan’s (2001) work on *compartmentalisation* provide a useful way of examining the spatial and structural invisibilities of migrants, and the practices that position them as outsiders. For it is within the tightly defined spaces of meat-processing factories and the social relations subsequently engendered in local communities across Wales, that discursive constructions of good/bad and moral/immoral come to the fore in ways that enhance the liminality of the migrant worker experience to position them as outsiders. Key findings from our research confirm Tannock’s (2013) argument that coalitions of employers and state agencies are able to reframe labour market conflict as a matter of local worker deficit and poor labour supply rather than poor job quality. As in Anderson’s (2000) account of domestic labour, our research points to how the structural and spatial invisibilities of migrant workers are directly linked to their conditions of employment and, more particularly, to those determined by the supply chain practices of major supermarkets. Our study does not draw on the same level of ethnographic insight as Anderson’s research, but it is clear that migrants working in meat-processing factories across Wales experience problems directly related to these spatial and structural invisibilities. Many leave a previous ‘fixed state’ to live on the margins with little chance of their rites of passage being fully consummated (Turner, 1979b). While they do not have the same non-status as the undocumented migrants discussed by Anderson (2000) or Chavez (1991), we conclude that some of the UK’s major economic players benefit from allowing them to remain ‘outsiders’.

## Notes

1. The Holocaust needs no explanation.

2. The Czech Republic, Estonia, Hungary, Latvia, Lithuania, Poland, Slovakia and Slovenia joined the EU in 2004.

3. Bulgaria and Romania joined in 2007.

4. Nationality was self-defined by interviewees.
